# Screening of the Promising Direct Thrombin Inhibitors from Haematophagous Organisms. Part I: Recombinant Analogues and Their Antithrombotic Activity In Vitro

**DOI:** 10.3390/biomedicines10010011

**Published:** 2021-12-22

**Authors:** Maria A. Kostromina, Elena A. Tukhovskaya, Elvira R. Shaykhutdinova, Gulsara A. Slashcheva, Alina M. Ismailova, Victor A. Palikov, Yuliya A. Palikova, Igor A. Dyachenko, Irina N. Kravchenko, Elena S. Sadovnikova, Nadezhda I. Novikova, Natalia A. Perepechenova, Evgeniy A. Zayats, Yuliya A. Abramchik, Dmitry D. Lykoshin, Andrey N. Mamaev, Elena V. Grigorieva, Andrey P. Momot, Arkady N. Murashev, Roman S. Esipov

**Affiliations:** 1 Laboratory of Biopharmaceutical Technologies, Shemyakin and Ovchinnikov Institute of Bioorganic Chemistry, Russian Academy of Sciences, Miklukho-Maklaya Street, 16/10, 117997 Moscow, Russia; eaz96post@gmail.com (E.A.Z.); ugama@yandex.ru (Y.A.A.); ldd-94@yandex.ru (D.D.L.); esipov@ibch.ru (R.S.E.); 2 Biological Testing Laboratory, Branch of Shemyakin and Ovchinnikov Institute of Bioorganic Chemistry, Russian Academy of Sciences, Pushchino, Prospekt Nauki, 6, 142290 Moscow, Russia; shaykhutdinova@bibch.ru (E.R.S.); slashcheva_ga@mail.ru (G.A.S.); ismailowa.a.m@yandex.ru (A.M.I.); vpalikov@bibch.ru (V.A.P.); yuliyapalikova@bibch.ru (Y.A.P.); dyachenko@bibch.ru (I.A.D.); ikravchenko@bibch.ru (I.N.K.); elenasadovnikova@yandex.ru (E.S.S.); nov_n@mail.ru (N.I.N.); murashev@bibch.ru (A.N.M.); 3 Laboratory of Toxicology In Vitro, Branch of Shemyakin and Ovchinnikov Institute of Bioorganic Chemistry, Russian Academy of Sciences, Pushchino, Prospekt Nauki, 6, 142290 Moscow, Russia; natka_1511@mail.ru; 4 Altai Branch of FSBI, National Research Center for Hematology, Ministry of Healthcare of the Russian Federation, 656045 Barnaul, Russia; amamaev@yandex.ru (A.N.M.); jeniagrigoriev@mail.ru (E.V.G.); xyzan@yandex.ru (A.P.M.)

**Keywords:** anticoagulant, direct thrombin inhibitor (DTI), haemadin, variegin, hirudin-1, anophelin, heparin, intein, coagulation time test

## Abstract

The success in treatment of venous thromboembolism and acute coronary syndromes using direct thrombin inhibitors has stimulated research aimed at finding a new anticoagulant from haematophagous organisms. This study deals with the comparison between hirudin-1 from *Hirudomedicinalis*(desirudin), being the first-known and most well-studied natural anticoagulant, along with recombinant analogs of haemadin from the leech *Haemadipsa sylvestris*, variegin from the tick *Amblyomma variegatum*, and anophelin from *Anopheles albimanus*. These polypeptides were chosen due to their high specificity and affinity for thrombin, as well as their distinctive inhibitory mechanisms. We have developed a universal scheme for the biotechnological production of these recombinant peptides as pharmaceutical substances. The anticoagulant activities of these peptides were compared using the thrombin amidolytic activity assay and prolongation of coagulation time (thrombin time, prothrombin time, and activated partial thromboplastin time) in mouse and human plasma. The preliminary results obtained suggest haemadin as the closest analog of recombinant hirudin-1, the active substance of the medicinal product Iprivask (Aventis Pharmaceuticals, USA) for the prevention of deep venous thrombosis in patients undergoing elective hip or knee replacement surgery. In contrast, variegin can be regarded as a natural analog of bivalirudin (Angiomax, The Medicines Company), a synthetic hirudin-1 derivative certified for the treatment of patients undergoing percutaneous coronary intervention and of patients with unstable angina pectoris after percutaneous transluminal coronary angioplasty.

## 1. Introduction

Disturbances in the blood coagulation system due to an imbalance of coagulation, fibrinolysis, and inflammation processes lead to the formation of blood clots in the vessels. Arterial thrombosis is the major cause of ischemic stroke and acute myocardial infarction, whereas venous thrombosis can lead to deep vein thrombosis (DVT), pulmonary embolism (PE), and unstable angina [1]. Major orthopedic surgery, including hip or knee replacement surgery or hip fracture surgery, are associated with a high risk of DVT/PE arterial and venous thrombosis. Targeting the components of both arterial and venous thrombi, antithrombotic drugs encompass antiplatelet agents, anticoagulants, and fibrinolytic drugs [2]. Therapy with a combination of various drugs is a necessary step in the treatment of arterial and venous thromboembolisms [3,4]. Direct and indirect anticoagulants are the basis for the prevention and treatment of venous thromboembolism (VTE) [5,6]. The former directly inhibit the serine proteases of the blood coagulation cascade, while the latter prevent the formation of precursors of prothrombin and other coagulation factors [7,8]. The main problem with any antithrombotic agent is that its administration strongly increases the probability of hemorrhagic complications. Therefore, the key criteria in the development of new anticoagulants include not only high efficiency and specificity, but also a safe pharmacological profile. The general problem of the antithrombotic therapy is the difficulty of striking the optimal balance between efficacy and safety, particularly with regard to bleeding.

Until recently, unfractionated heparin and warfarin (Coumadin, Bristol-Myers Squibb Company, New York, NY, USA) have been the mainstay therapy for venous thromboembolism [9]. However, complications of heparin therapy not only include hemorrhages, but also heparin-induced thrombocytopenia (HIT) and osteoporosis [10]. Therefore, when the probability of HIT is high, preference is given to a treatment with low-molecular-weight heparins and direct antithrombin III-independent thrombin inhibitors such as dabigatran etexilate (Pradaxa, Boehringer Ingelheim Pharma, GmbH & Co.KG), argatroban, and derivatives of hirudin-1, the natural thrombin inhibitor from the medicinal leech *Hirudomedicinalis* (bivalirudin, the brand name Angiomax, The Medicines Company and desirudin, the brand name Iprivask, Aventis Pharmaceuticals, etc.) [7,11,12,13]. Hirudin-1, and its recombinant derivative lepirudin (Refludan, Schering AG, licensed to Berlex laboratories, Inc., USA and Canada; Pharmion^®^, all other countries), was the first naturally occurring thrombin inhibitor to be approved by the European Agency for the Evaluation of Medicinal Products (EMA) and the US Food and Drug Administration (FDA) for the treatment of heparin-induced thrombocytopenia and associated thrombotic disease [14,15,16]. The efficacy and safety of both hirudin-1 and lepirudin were suggested for the prevention and treatment of VTE. However, frequent hemorrhagic adverse events (death, limb amputation, bleeding from puncture sites and wounds, intracranial bleeding, and major bleeding) were later observed in three large clinical trials, called the Heparin-Associated-Thrombocytopenia (HAT) 1,2, and 3, in patients treated with lepirudin [17]. Due to the results of these studies, the FDA narrowed the scope of lepirudin in 2000 [18]. Subsequently, lepirudin was discontinued from the market in 2012 [19]. However, the recombinant analogue of hirudin-1, desirudin (Revasc Canyon Pharmaceuticals, UK, or Iprivask, Aventis Pharmaceuticals, USA), was still approved for the prevention of deep venous thrombosis in patients undergoing elective hip or knee replacement surgery in a number of countries, with some restrictions [20]. Currently, synthetic bivalirudin (Angiomax, The Medicines Company, USA) is the most promising anticoagulant among hirudin-1 analogues in treatment of VTE [21,22,23]. Bivalirudin has been certified and used for a long time for treatment of patients with, or at risk of, heparin-induced thrombocytopenia (HIT) or heparin-induced thrombocytopenia and thrombosis syndrome (HITTS) undergoing percutaneous coronary intervention and of patients with unstable angina pectoris after percutaneous transluminal coronary angioplasty [24,25]. Although bivalirudin is not currently fully FDA-approved for use in pediatric patients, it is widely used for systemic anticoagulation during the therapy of pediatric patients requiring Extracorporeal Membrane Oxygenation (ECMO) [26,27,28]. The U.S. Food and Drug Administration (FDA) has granted the pediatric exclusivity for bivalirudin, based on the prospective, open-label, multi-center, single arm study submitted in response to a written request by the FDA to investigate the use of bivalirudin in pediatric patients aged birth to 16-year old [29]. Taking into account the significant differences in the level of development of the cardiac system in adult patients and children, bivalirudin shows a predictable and similar pattern of action [29,30].

This experience has stimulated research on various haematophagous organisms as a probable source of specific protein thrombin inhibitors [31]. Thus, a number of anticoagulants have been identified to date, and many of them have antithrombin activity similar to that of hirudin-1. Among the known natural inhibitors with high specificity and affinity for thrombin, we have selected the following peptides as the research subjects: haemadin from the Indian leech *Haemadipsa sylvestris*, variegin from the ixodid tick *Amblyomma variegatum*, and anophelin from the malaria mosquito *Anopheles albimanus*. All the above polypeptides are highly specific bivalent inhibitors of thrombin. It is known that thrombin is a multifunctional enzyme containing a typical serine protease active center and numerous binding sites for various substrates, including fibrinogen-binding exosite-1, heparin-binding exosite-2, and a number of accessory subsites [32]. Anophelin shows a unique reverse-binding mechanism of interaction with thrombin: it binds to the same sites as hirudin-1 (the active center and exosite-1) but in the reverse orientation [33]. In contrast, variegin blocks the active center and exosite-1 by the same mechanism as does hirudin-1 and hirulog-1 [34]. A specific feature of haemadin is that it binds to the thrombin active center and exosite-2, but in no way blocks the functionally important exosite-1 [35].

In our previous papers, we have reported the production of the recombinant hirudin-1 and anophelin, an anticoagulant from the malaria mosquito *Anopheles albimanus* [36,37]. According to the published data, solid-phase peptide synthesis is still the only effective method to produce variegin [38]. A recombinant analog of haemadin has been lab-scale produced by means of its periplasmic expression in *E. coli* as a fusion with maltose-binding protein, followed by its cleavage with factor Xa protease [39]. In the course of this study, we have developed the semipreparative biotechnological procedure for the intein-mediated production of recombinant haemadin and variegin with pharmaceutical purity, with the possibility of scaling up to large, industrial levels.

Our research is focused on the assessment of the potential of recombinant analogs of haemadin, anophelin, and varieginin in comparison with recombinant hirudin-1 using some of the classical methods for studying antithrombotic activities in vitro.

## 2. Materials and Methods

### 2.1. Chemicals, Enzymes, and Materials

Expression constructs were prepared using the plasmid vectors pAL2-T (Evrogen, Moscow, Russia), pTWIN1 (New England Biolabs, Ipswich, MA, USA), and pERIG [37], Encyclo DNA polymerase (Evrogen), restriction endonucleases NdeI, BamHI, and LguI, and T4 DNA ligase (Thermo Fisher Scientific, Burlington, ON, Canada). The proteins were chromatographically purified on XK 16/20 (lab-scale process), HiScale 50/40, and HiScale 50/20 columns (semi-prep scale process) with Q Sepharose XL and Q Sepharose HP resins (Cytiva, Danaher Corporation, Washington, DC, USA), a Kromasil 300-10-C18 RP-HPLC column (Kromasil, Nouryon, Bohus, Sweden), and BPG 140/750 column with Bio-Gel P-2 media (Bio-Rad Laboratories, Hercules, CA, USA). Thrombin (HYPHEN BioMed, Neuville-sur-Oise, France) and S-2238 (Chromogenix, Milan, Italy) were used for the determination of antithrombin activity. The thrombin-test, APTT-test, and thromboplastin from RPA «RENAM» (Moscow, Russia) were used in coagulation tests with mouse plasma. Thromborel-S, Pathromtin SL, and Test Thrombin reagents from Siemens Healthcare Diagnostic (Erlangen, Germany) were used in coagulation tests with human plasma. Recombinant hirudin-1 and anophelin were obtained according to the protocol published previously [36,37]. Other reagents used in this study were purchased from Sigma-Aldrich (St. Louis, MO, USA) and Panreac (Barcelona, Spain).

### 2.2. Bacterial Strains and Medium

Bacterial strain *Escherichia coli* One Shot™ TOP10 [F-*mcrA*Δ(*mrr-hsd*RMS-*mcr*BC) Φ80*lac*ZΔM15 Δ*lac*X74 *rec*A1 *ara*D139 Δ(*araleu*)7697 *gal*U*gal*K*rps*L (StrR) *end*A1 *nup*G] from Invitrogen (Waltham, Massachusetts, USA) was used as the host for recombinant plasmid construction and amplification. *E. coli* strains ER2566 [F^-^lamda^-^ fhuA2 [lon] ompTlacZ:: T7 gene1 gal sulA11 D(mcrC-mrr)114::IS10 R(mcr-73::miniTn10-Tet^S^)2 R(zgb-210::Tn10) (TetS) endA1 [dcm]] from New England Biolabs (Ipswich, MA, USA) and *E. coli* One Shot™ BL21(DE3) [F^–^*dcmompThsdS*(r_B_^–^m_B_^–^) *gal* λ (DE3)] from Invitrogen were used for protein expression. The culture medium for shake-flask cultivation was prepared using Bacto yeast extract, Bacto tryptone, and Bacto agar from Becton Dickinson and Company (Sparks, MD, USA). The culture medium for batch fermentation was prepared using casein peptone and yeast extract from A. Costantino & C.S.P.A. (Turin, Italy), as well as the silicone antifoaming agent PENTA 465 (Penta, Moscow, Russia).

### 2.3. Construction of Expression Vectors Containing the Haemadin and Variegin Genes

The haemadin (UniProtKB accession number Q25163) and variegin (UniProtKB accession number P85800) gene sequences were optimized for *E. coli* codon usage according to the Codon Usage Database (http://www.kazusa.or.jp/codon/, accessed on 7 December 2021) [40]. The haemadin and variegin genes were assembled from partially overlapping synthetic oligonucleotides (Table S1) and cloned into the plasmid vector pEL2-T.

The haemadin gene was subcloned into the original expression vector pERIG atLguI and BamHI restriction sites [37]. The variegin gene was subcloned into the expression vector pTWIN1 atNdeI and SapI restriction sites. The reading frames of the target fusion genes in vectors pERIG-Hae and pERVar-IG were verified by sequencing (Evrogen, Moscow, Russia).

### 2.4. Shake-Flask Fermentation of E. coli Producer Strains of Fusion Proteins GyrA-Hae and Var-GyrA

The expression vectors pERIG-Hae and pERVar-IG were used to transform the *E. coli* BL21 (DE3) strain. The transformed cells were plated on LB agar plates supplemented with ampicillin (100 µg/mL) and grown at 37 °C for 14 h to obtain separate colonies. Thereafter, several colonies were transferred to an Erlenmeyer flask with 100 mL of liquid LB medium containing 2% (*w*/*v*) glucose and ampicillin (100 µg/mL) and grown at 37 °C on a Certomat S II orbital shaker (Sartorius, Göttingen, Germany) at 180 rpm for 16 h to obtain an overnight culture with OD600 = 3.5. This culture (2% *v*/*v*) was used to inoculate 100 mL of LB medium with 0.2% (*w*/*v*) glucose and ampicillin (100 μg/mL), which were incubated on the shaker at 180 rpm and 37 °C for 6 h. This inoculum was used to prepare Working (WCB) and Master Cell Bank (MCB) for each strain for long-term storage at −80 °C.

The optimum growing conditions for each strain were determined in the small-scale cell cultivation in shake flasks. The WCB stock was plated on LB agar plates. Inoculum was prepared by transferring several colonies to a flask with 100 mL of liquid LB medium containing 2% (*w*/*v*) glucose and ampicillin (100 µg/mL) and grown at 37 °C for 6 h. This preculture (2% *v*/*v*) was inoculated to flasks with 150 mL of LB medium with 0.2% (*w*/*v*) glucose and ampicillin (100 µg/mL). When the cultures reached a density of OD600 = 0.8–0.9, they were supplemented with 0.4 mM IPTG and grew for another 4 h at 23 or 37 °C. The expression level of GyrA-Hae and Var-GyrA proteins was estimated by SDS-PAGE analysis (see below). The selected conditions were maintained in the course of shake-flask fermentation of the producer strain culture with a total volume of 1L. The cells were then pelleted by centrifugation at 3900× *g* for 20 min at 4 °C in an Avanti J-30I centrifuge (Beckman Coulter, Brea, CA, USA).

### 2.5. Purification and Cleavage of the GyrA-Hae and Var-GyrA Fusion Proteins

To isolate fusion proteins, we developed a unified laboratory-scale scheme based on anion-exchange chromatography. The 2 g of cell biomass were resuspended in 50 mM Tris-HCl buffer, pH 7.4, containing 5 mM EDTA and 1 mM PMSF in a ratio of 1:10 *w*/*v* and disintegrated by a Q700 Sonicator ultrasonic homogenizer (QsonicaLlc, Newtown, CT, USA) for 15 min at 4 °C (pulse-on time 2 s, pulse-off time 10 s, power 25 W, frequency 25 kHz). Cell debris was pelleted by centrifugation at 19,000× *g* for 20 min at 4 °C in a Hermle Z383K centrifuge (HERMLE Labortechnik GmbH, Wehingen, Germany). The supernatant was collected, diluted four-fold with 50 mM Tris-HCl buffer, pH 8.5, 5 mM EDTA, and filtered through membrane filters with a pore size of 0.45 μm (Millipore, Billerica, MA, USA). The filtered solution was applied onto an XK 16/20 column with 20 mLof Q Sepharose XL media equilibrated with 50 mM Tris-HCl buffer, pH 8.5, 5 mM EDTA. The target protein was eluted with a 15 CV linear gradient of 0 to 500 mM NaCl in 50 mM Tris-HCl buffer, pH 8.5, 5 mM EDTA with a flow rate of 60 cm/h. Chromatographic fractions were resolved by SDS-PAGE analysis, and those containing the target protein were pooled. The resulting solution was diluted with 50 mM Tris-HCl buffer, 5 mM EDTA, pH 9.0 to a final protein concentration of 0.5 ± 0.1 mg/mL.

Cleavage of the GyrA-Hae fusion protein was analyzed in the range pH 6.0 to 7.0 with step 0.2 at 23, 30, and 37 °C for 48 h. Cleavage of the Var-GyrA protein was analyzed at pH 8.5 at 23, 30 and 37 °C, in the presence of different DTT concentrations (5, 10, 25, and 50 mM) for 48 h. All experiments were performed in three replications. Here, and at subsequent stages, the results were evaluated by SDS-PAGE and protein measurements by the Lowry method [41].

### 2.6. Batch Fermentation

The batch fermentation of *E. coli* strains BL21(DE3)/pERIG-Hae and BL21(DE3)/pERVar-IG was carried out in a 75 L fermenter (Electrolux Fermentation, Novaferm, Falkenberg, Sweden) with the initial working volume of 50 L as described previously [37].

### 2.7. Semi-Preparative Purification of Haemadin and Variegin

The steps of purification and cleavage of the fusion protein were scaled up to develop a universal scheme of semi-preparative purification. A cell biomass weighing 100 g, obtained by the fed-batch fermentation, was disintegrated by sonication treatment in a Labsonic P ultrasonic homogenizer with aflow cell (Sartorius, Göttingen, Germany) for 90 min at 4 °C (pulse-on time 5 s, pulse-off time 10 s, power 50 W, frequency 25 kHz). The cell supernatant was obtained by centrifugation at 7500× *g* for 40 min at 10 °C in an Avanti J-30I centrifuge and was then diluted four-fold and adjusted to pH 8.5. The fusion protein was purified on an HiScale 50/40 column with 500 mL of Q Sepharose XL media as described above (15 CV linear gradient, flow rate of 150 cm/h).

For cleavage of the GyrA-Hae fusion protein, the eluate after anion-exchange chromatography (IEX) was adjusted to pH 6.0 and incubated at 37 °C for 48 h. For cleavage of the Var-GyrA fusion protein, the eluate after anion IEX was incubated with 50 mM DTT at 37 °C for 48 h. The cleavage efficiency was estimated by analytical RP-HPLC (see below).

Purification of the target peptide from the fusion protein cleavage mixture was performed using anion-exchange chromatography and reverse-phase HPLC (RP-HPLC). The solution containing products of the fusion protein cleavage was diluted five-fold with 10 mM MES buffer, pH 5.4, and adjusted to pH 5.1. Precipitated proteins were removed by centrifugation at 7500× *g* for 40 min at 10 °C and subsequent filtration through cellulose filters with a pore size of 4–12 μm (Macherey-Nagel, Düren, Germany). The filtrate was loaded onto a HiScale 50/20 column with 200 mL of Q Sepharose HP media equilibrated with 10 mM MES, pH 5.1, and the target peptide was eluted with a 10 CV linear pH gradient of 20 mM sodium acetate buffer with 50 mM NaCl, pH 4.8 and pH 3.6 with a flow rate of 150 cm/h. The material eluted from the column was resolved by analytical RP-HPLC, and fractions containing the target polypeptide were pooled.

At the next step, purification by RP-HPLC on a Kromasil 300-10-C18 column (particle size 10 μm, 250 × 50 mm) was performed. The pooled eluate after anion IEX was supplemented with acetonitrile and trifluoroacetic acid to final concentrations of 5% and 0.1%, respectively, and loaded onto the column. The material was eluted with an acetonitrile gradient of 6 to 50% in 0.1% TFA (8CV with a flow rate of 150 cm/h) and resolved by analytical RP-HPLC.

The purified peptide desalting was achieved by size-exclusion chromatography. Fractions containing the target peptide with a purity of above 99% after RP-HPLC were pooled and loaded onto a BPG 140/750 column with 7 L of Bio-Gel P-2 resin. The chromatography was performed in 5 mM ammonium bicarbonate buffer, pH8.0, with a flow rate of 20 cm/h. Fractions containing the target peptide were pooled and lyophilized in a VirTisAdVantage Plus XL Freeze Dryer/Lyophilizer (SP Scientific, Warminster, PA, USA).

### 2.8. Measurement of Antithrombin Activity of the Direct Thrombin Inhibitor In Vitro

#### 2.8.1. Inhibition of Thrombin Amidolytic Activity

Hirudin-1, anophelin, haemadin, and variegin were assayed for their abilities to inhibit human α-thrombin amidolytic activity toward of the specific chromogenic substrate S2238 (H-D-phenylalanyl-L-pipecolyl-L-arginine-*p*-nitroaniline dihydrochloride) [37]. The polypeptides were dissolved in a reaction buffer (50 mM Tris-HCl buffer, pH 8.4, with 150 mM NaCl and 0.1% PEG 6000) for a 1 mM stock solution, and subsequently diluted for different concentrations: 10 pM to 100 nM for haemadin and hirudin, 10 pM to 10 µM for anophelin and variegin. Assays were performed in 96-well microtiter plates using a Synergy HTX Multi-Mode Microplate Reader (Bio-Tek Instruments, Winooski, VT, USA) with a dual reagent injector module. The substrate and thrombin stock solutions were diluted in the reaction buffer.

In the tight-binding kinetic assays, all peptides were incubated with 500 pM thrombin (0.03 mNIH units) in a 100 μL mixture for 30 min at 37 °C and 300 rpm on an orbital thermo-shaker PST-60HL (Biosan, Riga, Latvia). The assays were carried out with varying inhibitor concentrations in triplicate: a range from 0.5pM to 10 nM (5 × 10^−4^–20 × [E_0_]) for hirudin and haemadin, or a range from 2.5 pM to 1 µM (5 × 10^−3^–2000× [E_0_]) for anophelin and variegin. The reactions were started with the programmed addition of the reaction buffer and chromogenic substrate (1 mM stock solution) by an injector in different ratios to a final substrate concentration of 50, 100, 200, 300, 400, or 500 µM to a final volume of 200 µL. The slow-binding assays were performed at constant substrate concentration (100 µM), and preincubated for 2 min at 37 °C with varying inhibitor concentrations (the same as in the tight-binding assays). The α-thrombin (500 pM) was added to a mixture to initiate the reactions. In both experiments, the plate with the reaction mixtures was incubated at 37 °C for 120 min and the rates of *p*-nitroaniline formation were measured at 405 nm.

#### 2.8.2. Kinetic Analysis of Thrombin Inhibition

The results of tight- and slow-binding assays were used to fit kinetic plots of *p*-nitroaniline accumulation as a function of the concentrations of the inhibitor and substrate. The dose–response curves of the percentage of thrombin inhibition at 120 min vs. the logarithmic function of the concentration of inhibitor at 100 µM of substrate were used to determine the half-maximal inhibitory concentration (IC50) values using OriginLab software version 9.1.0(OriginLab Corporation, Northampton, MA, USA) [38]. The velocity in the absence of inhibitor was considered as a reference point (0% inhibition).

The kinetic constants of thrombin inhibition were calculated in correspondence with the Morrison equation for tight-binding competitive inhibitors. Based on the dose–response curves of velocity as function of the concentration of the inhibitor, the apparent inhibitory constant Kiʹ was calculated using a non-linear regression analysis in OriginLab software. The resulting Kiʹ were plotted against the substrate concentration using linear regression to obtain the inhibitory constant Kiʹ.

#### 2.8.3. Coagulation Tests in Mouse Plasma

One hundred and two 7–8-week-old specific pathogen-free male ICR mice were obtained from the Laboratory Animal Breeding Facility «Pushchino» (BIBCh, Pushchino Branch of the Shemyakin and Ovchinnikov Institute of Bioorganic Chemistry of Russian Academy of Sciences). Blood was taken from the inferior vena cava under anaesthesia (telazol–xylazine in 1:2 *v*/*v* ratio) using a blood collection tube containing citrate in a 9:1 ratio (*v*/*v*, plasma/citrate). The plasma was obtained after centrifugation of the blood-citrate mixture for 15 min at 1600× *g* at 23 °C.

Each peptide was dissolved in PBS to a concentration of 1 mg/mL, and then diluted with PBS to three stock concentrations: 1, 10, and 100 µg/mL. All compounds tested were mixed with the pooled plasma in a 9:1 ratio at 23 °C immediately prior the experiment to test concentrations: 0, 0.1, 1, 10, and 100 µg/mL. The thrombin time (TT), the activated partial thromboplastin time (APTT), and the prothrombin time (PT) tests were performed using the Thrombin-test, APTT-test, and thromboplastin from RPA «RENAM» (Moscow, Russia) according to the reagent manufacture’s guidance. All measurements were performed within 3 min at least 5 times using the coagulometer CL 4, (BehnkElektronik, Norderstedt, Germany).

#### 2.8.4. Coagulation Tests in Human Plasma

The venous blood was collected from healthy volunteers into Vacuette blood collection tubes with sodium citrate (9NC Sodium citrate, 3.2%, 4 mL, Greiner Bio-One GmbH, Austria) in a 9:1 ratio (*v*/*v*, plasma/citrate). The blood samples were centrifuged at 1400× *g* for 40 min at 23 °C, pooled, aliquoted, and stored for 3 days at −40 °C until analysis. The pooled plasma was used immediately or for up to 3 h after thawing at 37 °C.

Each peptide was dissolved in a 50 mM Tris-HCl buffer, pH 7.4, to 1 mg/mL and then diluted to different concentrations (0.1, 1, 10, and 100 µg/mL) at 23 °C and mixed with the pooled plasma in a 9:1 ratio immediately prior to the experiment to test the concentrations of 0, 0.01, 0.1, 1, and 10 µg/mL. The TT, APTT, and PT tests were performed according to the reagent manufacture’s guidance. All measurements were performed within 3–4 min at least 5 times using the coagulometer Start 4 (DiagnosticaStago, Paris, France).

### 2.9. Analytical Methods

Sodium dodecyl sulfate-polyacrylamide gel electrophoresis (SDS-PAGE) was performed according to Laemmli [37,42]. Analytical reverse-phase high-performance liquid chromatography (RP-HPLC) and mass spectrometry analysis were carried out as described previously [37].

### 2.10. Statistical Analyses

Statistica for Windows and PRISM version 8 software were used for statistical analysis. The results are expressed as means ± SD. The significance of differences in multiple comparisons was determined using a one-way ANOVA, Kruskal-Wallis test and Mann–Whitney U-test. Significance level was determined at *p* ≤ 0.05.

## 3. Results

### 3.1. Recombinant Haemadin and Variegin Production

#### 3.1.1. Construction of Expression Vectors

To produce haemadin from the land-living leech *Haemadipsa sylvestris* and variegin from the bont tick *Amblyomma variegatum*, we used a technology based on the self-cleaving N- and C-terminal intein fusion tag (Figure S1). Haemadin is a 57-aa polypeptide with a molecular weight of 6251.88 Da (NCBI/Protein database accession number CAA79672) (Figure S1a). To produce the haemadin, we have used the original pH-dependent C-terminal cleavage construct based on the GyrA mini-intein from *Mycobacterium xenopi* (Figure S1b) [37]. Variegin is a 32-aa peptide with a molecular weight of 3607.94 Da (NCBI/Protein database accession number P85800) (Figure S1a). Variegin contains an N-terminal serine residue, and this interferes with its production by the means of a C-terminal cleavage construct, because the intein affinity tag easily cleaves off such exteins during the producer strain cultivation [43]. Hence, we have used a thiol-induced N-terminal cleavage construct based on the *Mxe*GyrA mini-intein for this purpose (Figure S1c).

The haemadin and variegin synthetic genes were constructed using chemical–enzymatic synthesis and cloned into pERIG and pTWIN1 plasmid vectors to produce the expression vectors pERIG-Hae and pERVar-IG, respectively (Figure S1c,d, Table S1).

#### 3.1.2. Cultivation of Strains Producing the Fusion Proteins Containing Haemadin and Variegin

The expression vector pERIG-Hae encoded the GyrA-Hae protein (35.4 kDa) consisting of haemadin fused to the C-terminus of CBD-tagged *Mxe*GyrA mini-intein. The pERVar-IG expression vector encoded the Var-GyrA protein (31.6 kDa), consisting of variegin fused to the N-terminus of CBD-tagged *Mxe*GyrA mini-intein.

We have evaluated the accumulation of the fusion proteins GyrA-Hae and Var-GyrA in transformed *E. coli* BL21(DE3) strains during 6 h of cultivation in shake flasks at 23 or 37 °C after induction with IPTG. We compared the levels of fusion protein biosynthesis (Figure S2). Cultivation of the producer strains for 4 h at 37 °C was the optimal condition for fusion protein accumulation. The induction stage extension up to 5 or 6 h did not contribute to a significant increase of protein accumulation. Cultivation of the producer strains was carried out with a total volume of 1 L. The relative expression level of the fusion protein and cell biomass yield reached 27.84 ± 0.69% and 5.6 ± 0.2 g/L for GyrA-Hae and 30.18 ± 0.52% and 5.8 ± 0.3 g/L for Var-GyrA under these conditions.

#### 3.1.3. Purification of Haemadin

**Figure 1:**
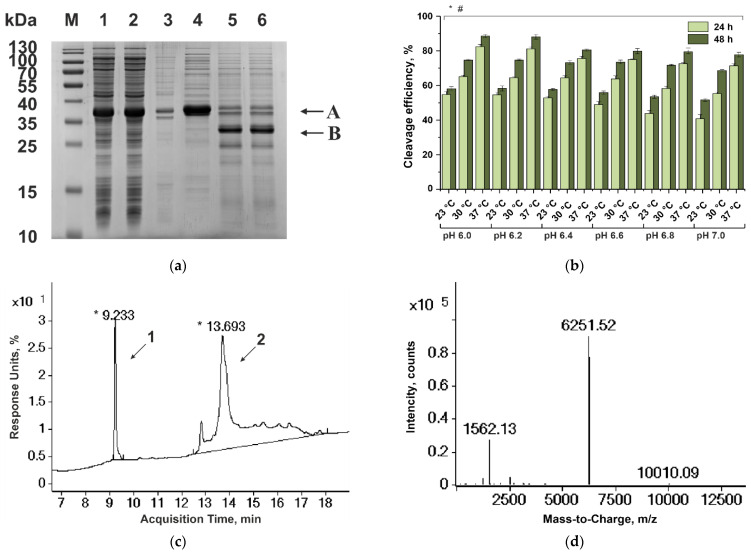
Purification of haemadin. (**a**) SDS-PAGE analysis of the purification and cleavage of the GyrA-Hae fusion protein. M, molecular mass markers; lane 1, crude cell lysate; lane 2, cell supernatant; lane 3, cell debris; lane 4, purified GyrA-Hae fusion protein after anion IEX; lanes 5–6, products of fusion protein cleavage at pH 6.0 and 37 °C for 24 h (lane 5) and 48 h (lane 6). Arrows indicate theGyrA-Hae fusion protein (A) and residual protein CBD-GyrA (B). (**b**) Diagram of GyrA-Haefusion protein cleavage in the range pH 6.0 to 7.0 at 23, 30, and 37 °C for 24 and 48 h. * indicates *p* ≤ 0.05 for the comparison of incubation time between 24 and 48 h, according to a Mann–Whitney test; # indicates *p* ≤ 0.05 for the comparison of incubation temperature between 23 and 37 °C, according to Kruskal–Wallis test. (**c**) RP-HPLC analysis of GyrA-Haefusion protein cleavage for 48 h at pH 6.0 and 37 °C. Arrows indicate haemadin (*1*) and residual protein CBD-GyrA (*2*). RP-HPLC in YMC-Pack C8 (Octyl) column (30 nm, 3 μm, 150 × 2.1 mm), elution with an acetonitrile gradient (8–64%) in 0.1% TFA at a rate of 0.3 mL/min. (**d**) ESI-TOF mass spectrum of haemadin.

Similar to hirudin-1, haemadin contains three intramolecular disulfide bonds. In the course of producer strain *E. coli* BL21(DE3)/pERIG-Hae cultivation, the GyrA-Hae fusion protein was expressed in a soluble form, which is an indirect piece of evidence for the correct formation of these bonds in haemadin. The GyrA-Hae protein was enriched by anion-exchange chromatography from the supernatant fraction obtained by the destruction of 5 g of cell biomass (Figure 1a). Our previous experiments showed that the highest level of its C-terminal cleavage for *Mxe*GyrA-based fusion proteins was achieved upon 48 h incubation at pH 6.0, above 30 °C, and reached in the presence of a thiol reagent [37]. In the case of the GyrA-Hae fusion protein, we refrained from using such a thiol-containing reagent, because this could result in the reduction of apparently correct disulfide bonds in haemadin. We analyzed the fusion protein cleavage in the range pH 6.0 to 7.0, with step 0.2 at 23, 30, and 37 °C for 48 h (Figure 1b and Figure S3). The highest cleavage efficiency was recorded after 48-h incubation at pH 6.0 or 6.2; the cleavage level reached 58.20 ± 1.51% at 23 °C (mean ± SD value between pH 6.0 and 6.2), 74.73 ± 0.50% at 30 °C, and 85.73 ± 1.35% at 37 °C. The target polypeptide after cleavage had a molecular weight of 6251.52 Da, precisely coinciding with the theoretical calculation for haemadin with three disulfide bonds (Figure 1c,d).

The fermentation of *E. coli* strain BL21 (DE3)/pERIG-Hae was scaled up from 300 mL shake flasks to a 75 L industrial fermenter. As a result of 50 L batch fermentation, 780 g of wet cell biomass (15.6 ± 0.8 g/L) was obtained. The relative content of the GyrA-Hae fusion protein was 27.80 ± 1.23% of the total cell protein. The developed protocol allowed to isolate haemadin from 100 g of cell biomass per production cycle. After purification by anion-exchange chromatography, the fusion protein was cleaved at selected conditions (48 h at pH 6.0 and 37 °C) with stirring at 300 rpm. Such a technological approach accelerated the dynamic of fusion protein cleavage: 82.30% efficiency was achieved after 24 h incubation and reached up to 86.90% after 48 h. At the next step, the procedure involved anion IEX with a pH gradient, RP-HPLC, and SEC was used to purify haemadin from the uncleaved fusion protein, CBD-tagged mini-intein, and cell proteins. This semi-preparative scheme allowed us to isolate 497 mg of haemadin with pharmaceutical purity (above 98.0%) from 100 g of cell biomass (77.5 mg protein per liter of cell culture; Table S2). The actual yield reached 68.57% of that theoretically possible.

#### 3.1.4. Purification of Variegin

**Figure 2:**
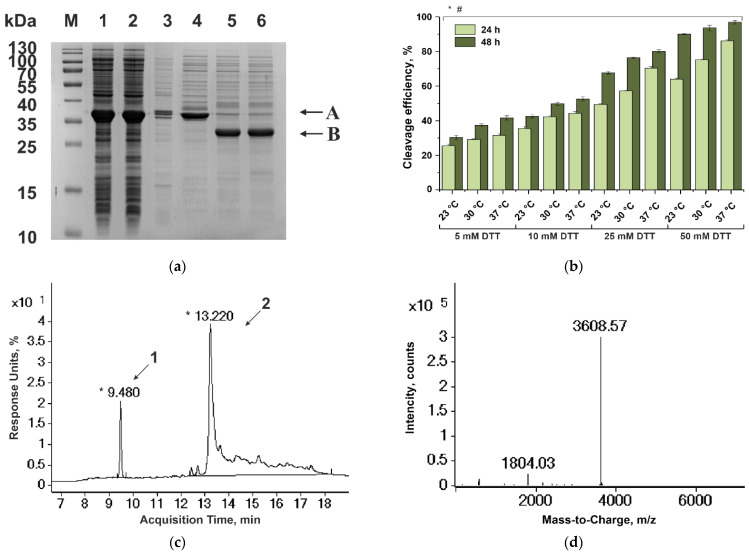
Purification of variegin. (**a**) SDS-PAGE analysis of purification and cleavage of Var-GyrA fusion protein. M, molecular mass markers; lane 1, crude cell lysate; lane 2, cell supernatant; lane 3, cell debris; lane 4, purified Var-GyrAfusion protein after anion IEX; lanes 5–6, products of fusion protein cleavage at pH 8.5 and 37 °C with 50 mM DTT for 24 h (lane 5) and 48 h (lane 6). Arrows indicate theVar-GyrA fusion protein (A) and residual protein CBD-GyrA (B). (**b**) Diagram of Var-GyrAfusion protein cleavage at pH 8.5 and 23, 30, and 37 °C for 24 and 48 h in the presence of 5, 10, 25, and 50 mM DTT. * indicates *p* ≤ 0.05 for the comparison of incubation time between 24 and 48 h according to a Mann–Whitney test; # indicates *p* ≤ 0.05 for the comparison of incubation temperature between 23 and 37 °C according to a Kruskal–Wallis test. (**c**) RP-HPLC analysis of Var-GyrAfusion protein cleavage for 48 h at pH 8.5 and 37 °C with 50 mM DTT. Arrows indicate variegin (*1*) and residual protein CBD-GyrA (*2*). RP-HPLC in Y.C-Pack C8 (Octyl) column (30 nm, 3 μm, 150 × 2.1 mm), elution with an acetonitrile gradient (8–64%) in 0.1% TFA at a rate of 0.3 mL/min. (**d**) ESI-TOF mass spectrum of variegin.

The Var-GyrA fusion protein synthesized by the *E. coli* producer strain BL21(DE3)/pERVar-IG in a soluble form was enriched from the supernatant fraction obtained by the destruction of 5 g of cell biomass (Figure 2a). It is known that N-terminal cleavage of the intein-tag from the fusion protein is most effective under medium alkaline conditions and in the presence of a thiol reagent [44]. We have analyzed Var-GyrA fusion protein cleavage at pH 8.5 in the presence of DTT (5, 10, 25, and 50 mM) at 23, 30, and 37 °C (Figure 2b and Figure S4). The highest cleavage efficiency (96.92 ± 1.03%) was achieved upon incubation for 48 h at pH 8.5, at 37 °C, and with 50mM DTT. Incubation at lower DTT concentrations and at other temperature was less effective. Thus, the optimal cleavage conditions for Var-GyrA have proved to be the same as those recommended by NEB for N-terminal cleavage of such fusion proteins.

The target cleavage product had a molecular weight of 3608.57 Da, which coincides with that of variegin without N-terminal formylmethionine (Figure 2c,d). The absence of N-formyl-Met-variegin (3739 Da) among cleavage products can be explained by the high efficiency of N-terminal processing in vivo of proteins containing serine in position P1’ [45].

The fermentation of the *E. coli* strain BL21(DE3)/pERVar-IG was scaled up from 300 mL shake flasks to a 75 L industrial fermenter. As a result of 50 L fed-batch fermentation, 745 g of cell biomass (14.9 g/L) was obtained. The expression level of Var-GyrA fusion protein was 32.8 ± 0.6% of the total cell protein. For the purification of variegin, we used the protocol developed for haemadin isolation. After purification by anion-exchange chromatography, the fusion protein was cleaved at selected conditions (48 h at pH 8.5, 37 °C, with 50 mM DTT) with stirring at 300 rpm. The cleavage efficiency reached 92.3% for 24 h and 93.9% for 48 h. Thus, the fusion protein cleavage stage was optimized and accelerated, as it was with the haemadin purification. Subsequent purification of variegin involved the same three-stage procedure as was used in the case of haemadin.

This semi-preparative scheme allowed us to isolate 189 mg of variegin with pharmaceutical purity (above 98%) from 100 g of cell biomass (28.2 ± 3.6 mg protein per liter of cell culture; Table S4). The actual yield reached 72.02% of that theoretically possible.

### 3.2. Antithrombin Activity

#### 3.2.1. Inhibition of the Amidolytic Thrombin Activity

**Figure 3:**
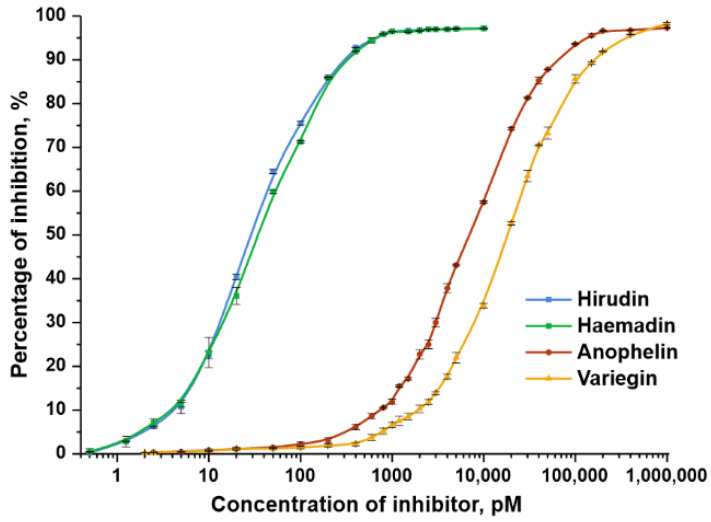
Dose–response curve of inhibition of amidolytic thrombin activity by hirudin-1, haemadin, anophelin, and variegin. A tight-binding assay was performed with 500 pM thrombin, 100 μM S-2238, and different concentrations of the inhibitor.

Hirudin-1, anophelin, haemadin, and variegin are bivalent, tight-binding competitive inhibitors of thrombin [33,34,35,46]. Their antithrombin activities were evaluated using the amidolytic test, and the results were used to plot the dose–response curve of thrombin inhibition as a function of inhibitor concentration (Figure 3) [37].

The values for IC50 and inhibition constants, *K*_i_, that were calculated are presented in Table 1. The affinity of each peptide increased, in a non-statistically significant manner, after incubation with thrombin, which is typical for fast, tight-binding inhibitors (Figure S5). The values for hirudin-1 and anophelin were similar to those determined previously [36]. The values calculated for haemadin and variegin coincide with those estimated by other authors [37]. As expected, haemadin proved to be most similar to hirudin-1 in terms of antithrombin activity.

#### 3.2.2. Results of Clotting Assays on Mouse and Human Plasma

The anticoagulation activity of hirudin-1, anophelin, haemadin, and variegin was estimated by activated partial thromboplastin time (APTT), prothrombin time (PT), and thrombin time (TT) clotting assays performed in vitro in mouse and human plasma. Interspecies differences in the coagulation profile between human and mice are a well-known fact that is a significant limitation in the interpretation of results obtained in coagulation experiments with mice or rats and humans [47,48]. In most studies, the effective concentration of antithrombotic agents for mice can be one to two orders of magnitude higher than the effective dose in humans. Its effects are due of different levels of coagulation and the fibrinolytic system reactivity. Conversion of human doses to animal equivalent doses is based on body surface area. For simplicity of recalculation, we used the 10× conversion factor for recalculation doses (Dm/Dh = 10) [49].

**Figure 4:**
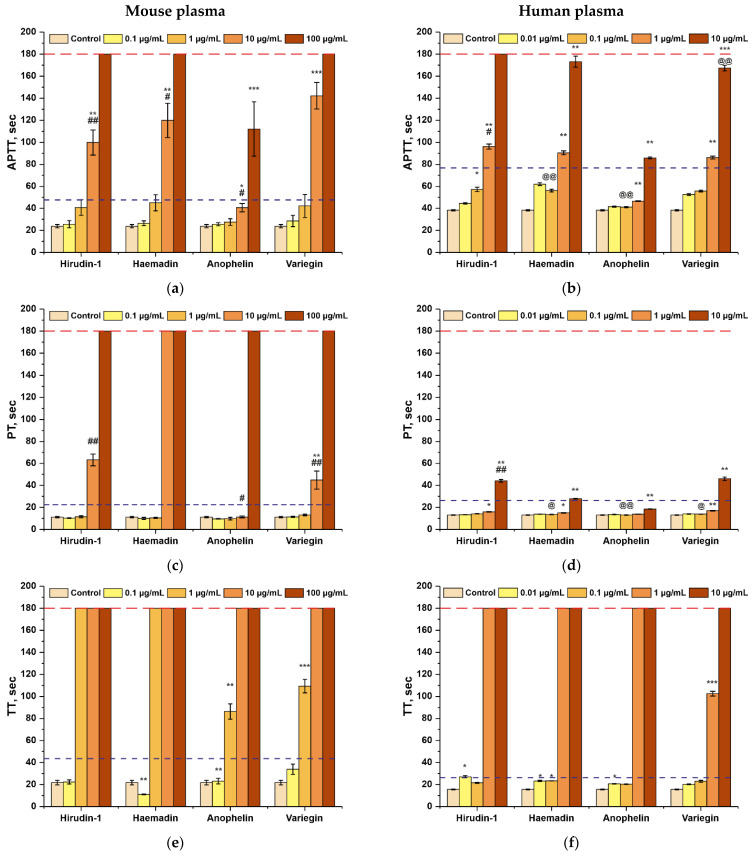
Results of the coagulation assays in mouse (**a**,**c**,**e**) and human (**b**,**d**,**f**) plasma. Effect of hirudin-1, anophelin, haemadin, and variegin on activated partial thromboplastin time (APTT) (**a**,**b**), prothrombin time (PT) (**c**,**d**), and thrombin time (TT) (**e**,**f**). All experiments were performed in five replications. Data are presented as the mean per group ± standard deviation. *** indicates *p* ≤ 0.001 compared to a Control (Saline), ** indicates *p* ≤ 0.01 compared to a control, and * indicates *p* ≤ 0.05 compared to a control according to a Kruskal–Wallis test; ## indicates *p* ≤ 0.01 compared to a concentration of 0.1 µg/mL (for mice) and 0.01 µg/mL (for humans) and # indicates *p* ≤ 0.05 compared to a concentration of 0.1 µg/mL (for mice) and 0.01 µg/mL (for humans) according to a Kruskal–Wallis test; @@ indicates *p* ≤ 0.01 compared to a concentration of 100 µg/mL (for mice) and 10 µg/mL (for humans) and @ indicates *p* ≤ 0.05 compared to a concentration of 100 µg/mL (for mice) and 10 µg/mL (for humans)according to a Kruskal–Wallis test. The blue dotted line indicates a 50% increase in the value relative to the control (saline). The red dotted line indicates the time limit for measuring the values of coagulation parameters (180 s).

All substances demonstrated a dose-dependent prolongation of the coagulation time that was similar between human and mouse blood plasma, taking into account the correlation coefficient. The effect of all peptides on internal pathways of coagulation activation (APTT test) was statistically significant (almost 2-fold prolongation), but only at concentrations of more than 1 µg/mL (in mouse plasma) or 10 µg/mL (in human plasma)—more than 150 nM of hirudin-1 and haemadin, 1.5 nM of anophelin, and 270 nM of variegin (Figure 4a,b, Tables S4–S9). Activation of the external pathways of coagulation (PT test) was achieved at concentrations of more than 10 μg/mL(more than 1.5 nM of hirudin-1 and haemadin, 15 nM of anophelin, and 2.7 nM of variegin). The dose-dependent effect of hirudin-1, anophelin, haemadin, and variegin on the thrombin clotting time (TT test) prolongation in mouse and human plasma was equal at the same doses, considering the 10× conversion factor.

## 4. Discussion

The blood coagulation cascade is a complex system of sequential reactions of enzyme activation with various negative and positive feedback loops, including a thrombin-mediated feedback loop [50,51]. Thrombin is involved in a wide variety of biological processes. It is known that through exosite-1, thrombin interacts with its partners biological proteins such as fibrinogen, fibrin, thrombomodulin, and the thrombin receptor. Through exosite-2,thrombin interacts with heparin, heparin cofactor II, antithrombin III, the kringle-2 domain in prothrombin, and platelet glycoprotein Ib (Gp1b) [52]. Therefore, the binding of inhibitors to these sites simultaneously blocks the active site, affecting not only the final step of the blood coagulation cascade (fibrin clot formation), but also other thrombin-mediated processes and activation of the feedback loop. For example, both exosites are involved to varying degrees in the proteolytic activation of FV, which takes partin the formation of the prothrombinase complex required for the amplification of the coagulation pathway [53]. It is exosite-1 that plays a critical role in the recognition of the cleavage site in FV, and if it is blocked, it significantly affects the initiation of FV activation. The interaction with exosite-2 takes place only during the final stage of FV activation, and by blocking its natural ligands, prothrombin fragment 2 and heparin, it only slightly affects FV activation with a large excess of the effectors. A similar situation can be observed with the role of exosites in the activation of the FVIII/thrombin feedback loop [54]. Due to the complex allosteric regulatory mechanisms triggered by the binding of effectors to one or another thrombin site, it is not possible to predict the consequences of the interaction accurately and clearly identify the processes that are triggered by the inhibition. For example, dabigatran etexilate and argatroban are highly selective active site inhibitors. However, dabigatran etexilate inhibits the binding of exosite-1 to γA/γA-fibrin or factor Va, and does not in any way affect the interaction of exosite-2 with Gp1b [55]. At the same time, argatrobanenhances the interaction of thrombin with all these effectors. The communication between exosites caused by the effector (inhibitor or nature partner) binding was described for the activation of protein C, a natural anticoagulant: binding of ligands to exosite-2 attenuated the exosite-1-mediated binding of thrombin to thrombomodulin [56].

The key challenge in the development of new anticoagulants is to strike the right balance between efficiency and safety. Medicines satisfying this criterion are known to have a better safety profile and a wider therapeutic dosage range. The interest in new highly specific thrombin inhibitors from blood-sucking animals and insects can be explained by the success in antithrombotic therapy with the derivatives of hirudin-1 from *Hirudomedicinalis*. Thus, the hirudin-1-based drug desirudin, or Iprivask (Canyon Pharmaceuticals, Inc.), is used to prevent proximal deep vein thrombosis in patients undergoing total hip or knee arthroplasty [23]. Bivalirudin is an active ingredient of medical drug Angiomax (The Medicines Company), approved for the therapy of patients with heparin-induced thrombocytopenia [24].

Hirudin-1isthe first-known and most well-studied natural bivalent thrombin inhibitor withinhibitions constants of about 200 fM.The structure of hirudin-1 includes a [1,2,3,4,5,6,7,8,9,10,11,12,13,14,15,16,17,18,19,20,21,22,23,24,25,26,27,28,29,30,31,32,33,34,35,36,37,38,39,40,41,42,43,44,45,46,47]-core domain that binds to the thrombin active site and unstructured [55,56,57,58,59,60,61,62,63,64,65]-tail that interacts with the fibrinogen-binding exosite-1 of thrombin [57]. As isolated peptides, both of thesefragments inhibited thrombin-mediated fibrinogen cleavage and clot formation, but with significantly reduced efficiency compared with the full molecule (N- and C-terminal fragments inhibit a fibrin clot formation with a Ki value of about 0.1–0.7 μM) [58]. Thrombin can cleave hirudin-1 at three Lys-X bonds (two bonds are located inside the N-terminal core and the other is situated between of the N-terminal domain and C-terminal tail) and thus inactivate the inhibition. The C-terminal [53,54,55,56,57,58,59,60,61,62,63,64]-fragment of hirudin-1 blocks the fibrinogen clotting activity of thrombin with a Ki of 0.15 μM and was used for the construction of the bivalent thrombin inhibitor, hirulog-1 or bivalirudin with a Ki value of 1.3 nM. It was fused with a synthetic N-terminal sequence (D-Phe)-Pro-Arg-Pro that tightly blocks the thrombin active site through a polymeric linker of four glycyl residues [59]. Nevertheless, due to the presence of the Arg-Pro bond, bivalirudin is quickly cleaved by thrombin and its antithrombotic effect is eliminated [60].

All of the polypeptides in our research paper—haemadin from the leech *Haemadipsa sylvestris*, variegin from the tick *Amblyomma variegatum*, and anophelin from the mosquito *Anopheles albimanus*—are highly specific, tight-binding thrombin inhibitors with unique structural and functional properties, and hold promise for antithrombotic therapy. Haemadin, with a Ki value of about 200 fM, has a similar mechanism of inhibition of thrombin and binds to the active center and exosite-2, but does not affect its exosite-1-dependent biological functions [39,61]. This is related to alack of the ability of hamaedin to inhibitan important active intermediateof thrombin—meizothrombin— and, accordingly, the functions that it performs [62,63]. Haemadin also was cleaved by thrombin at the Lys42-Ile bond, but the resulting fragment remains a competitive thrombin inhibitor with a Ki value of 5nM [61]. Anophelin is a cysteine-less bivalent thrombin inhibitor with a Ki value of about 30–100 pM, but with a unique inhibition mechanism: it binds to the active center and exosite-1, but in a reverse orientation [33,64]. The [32,33,34,35,36,37,38,39,40,41,42,43,44,45]-fragment block of the thrombin exosite-1 and the [50,51,52,53]-tetrapeptide interact with its active site. Although a 31 aa N-terminal fragment of anophelindoes not possess the antithrombin activity, due to its negative charge it can promote the correct formation of the complex with thrombin. The Lys/Arg-X thrombin cleavage sites in anophelin are located outside of its functional areas, which means that one can expect the thrombin-mediated inactivation to be insignificant. Variegin (32 aa) is the smallest among the naturally occurring bivalent thrombin inhibitors identified to date [38]. Natural variegin is characterized by high specificity and tight binding of thrombin (*K*_i_ = 10.4 pM). Unlike natural variegin, its synthetic analog (s-variegin) is not glycosylated at Tyr14, which results in a 30-fold decrease in its affinity for thrombin (antiamydolitic activity with a *K*_i_value increasing to 300 pM). Nevertheless, s-variegin, in this respect, is at least 20 times superior to conventional anticoagulants such as bivalirudin (*K*_i_ = 2.9 nM), argatroban (*K*_i_ = 3.2 nM), and dabigatran etexilate (*K*_i_ = 4.5 nM) [38,65]. In contrast to bivalirudin, variegin remains as a fast, tight-binding thrombin inhibitor, but with a non-competitive mechanism and a Ki of 14 nM after thrombin-mediated cleavage [34]. Therefore, a more prolonged action should be expected for variegin. The high degree of homology between the C-terminal fragments of bivalirudin and variegin, their comparable molecular size, and similar bivalent mechanism of thrombin inhibition suggest that their pharmacological and immunogenicity characteristics are similar.

The study of antithrombotic activity in vitro is only the first step in comparing the selected anticoagulants. The amidolytic test allows only for the evaluation of the affinity of peptides for thrombin and does not consider any additional effects. The thrombin time test also reflects the difference in the affinity of all peptides for thrombin. The results obtained are expected and correlate well with the results of the amidolytic test. However, the results of the APTT and PT tests are unexpected and difficult to explain without studying the molecular mechanisms of the action of the inhibitors. The determination of APTT is the main method for assessing the activation of the internal blood coagulation pathway because it allows for the assessment of the deficiency of factors I, II, V, VIII, IX, X, XI, and XII [51]. This method is mainly used to study direct thrombin inhibitors. The PT test reflects the triggering of the external blood coagulation pathway and is used to diagnose deficiencies of factors I, II, V, VII, and X, and to study indirect thrombin inhibitors (vitamin K-dependent group) [51]. It is especially worth noting that variegin, while being a significantly weaker inhibitor of thrombin in comparison with hirudin-1, showed practically the same results in the same effective concentrations. Perhaps this can be explained by the absence of thrombin-mediated inactivation of variegin. The fact that anophelinshowed itself as the weakest inhibitor in these tests [5] cannot be explained solely by the peculiarities in the mechanism of interaction with thrombin. It is possible that, unlike other inhibitors, anophelin does not trigger internal anticoagulation mechanisms.

Thus, haemadin and variegin have unique features, including a high anti-thrombin activity in vitro, and can be regarded as promising anticoagulants for clinical use, along with hirudin-1 and bivalirudin. Special attention should be paid to the absence of a correlation between the direct antithrombin activity of variegin and its effect on coagulation processes. «Small but effective» direct thrombin inhibitors such as variegin can take a place among contemporary anticoagulants as the closest (but much more active) natural analog of bivalirudin.

## 5. Conclusions

The preliminary results obtained show haemadin as an analog of recombinant hirudin-1 and variegin as a bivalirudin, a synthetic hirudin-1 derivative certified for treatment of patients with heparin-induced thrombocytopenia. However, the antithrombotic potential and safety profile can be adequately evaluated only in the course of in vivo studies on different models of the venous thromboses.

## Figures and Tables

**Table 1:** Parameters of antithrombin activity of hirudin-1, haemadin, anophelin, and variegin.

Thrombin Inhibitor	Preincubation Time	IC50 *	Inhibition Constant K_i_
Hirudin-1	0 min	21.47 ± 0.49 pM	
	30 min	28.01 ± 0.36 pM	217 ± 25 fM
Haemadin	0 min	22.03 ± 0.57 pM	
	30 min	33.15 ± 0.68 pM	237 ± 25 fM
Anophelin	0 min	6.35 ± 0.14 nM	
	30 min	6.78 ± 0.04 nM	99.4 ± 11.54 pM
Variegin	0 min	14.55 ± 1.88 nM	
	30 min	18.39 ± 0.61 nM	371.6 ± 22.5 pM

* indicates *p* ≤ 0.05 for IC50 values relative to 30 min incubation values according to a Kruskal–Wallis test.

## Supplementary Materials

The following are available online at https://www.mdpi.com/article/10.3390/biomedicines10010011/s1, Table S1. Oligonucleotide primers used to construct the artificial haemadin and variegin genes; Figure S1. (a) Amino acid sequences of haemadin and variegin. (b) Construction scheme of the expression vector pERIG-Hae. (c) Construction scheme of the expression vector pERVar-IG. (d) GyrA-Hae and Var-GyrA fusion proteins. CBD, chitin-binding domain; *Mxe*GyrA, mini-intein from *Mycobacterium xenopi*; A, alanine; H, histidine; N, asparagine; C, cysteine. Figure S1. (a) Amino acid sequences of haemadin and variegin. (b) Construction scheme of the expression vector pERIG-Hae. (c) Construction scheme of the expression vector pERVar-IG. (d) GyrA-Hae and Var-GyrA fusion proteins. CBD, chitin-binding domain; *Mxe*GyrA, mini-intein from *Mycobacterium xenopi*; A, alanine; H, histidine; N, asparagine; C, cysteine. Figure S2.Optimization of cultivation conditions of the producer strains *E. coli* BL21(DE3)/pERIG-Hae (a,b) and *E. coli* BL21(DE3)/pERVar-IG (c,d). (a,c) The SDS-PAGE analysis of the fusion proteins GyrA-Hae (a) and Var-GyrA (c) accumulation during the producer strain cultivation. M, molecular mass markers; Lane 1, uninduced crude cell extract; lanes 2–7, crude cell extracts after induction for 1–6 h at 23 °C; lanes 8–13, crude cell extracts after induction for 1–6 h at 37 °C. 15%-SDS-PAGE (b,d). Dynamics of the fusion proteins GyrA-Hae (b) and Var-GyrA (d) accumulation during the producer strains cultivation. * indicates *p* ≤ 0.05 relative to 37 °C according to a Mann–Whitney U test. Figure S3. Optimization of the GyrA-Hae fusion protein cleavage. (a,c,e) cleavage after 24-h incubation in the range pH 6.0 to 7.0 at 23 °C (a), 30 °C (b), and 37 °C (e). (b,d,f) cleavage after 48-h incubation in the range pH 6.0 to 7.0 at 23 °C (b), 30 °C (d), and 37 °C (f). M, molecular mass markers; lane 1, purified GyrA-Hae after anion IEX; lane 2, cleavage at pH 6.0; lane 3, cleavage at pH 6.2; lane 4, cleavage at pH 6.4; lane 5, cleavage at pH 6.6; lane 6, cleavage at pH 6.8; lane 7, cleavage at pH 7.0. 15%-SDS-PAGE. Arrows indicate the GyrA-Hae fusion protein (A) and residual protein CBD-GyrA (B). Figure S4. Optimization of the Var-GyrA fusion protein cleavage at pH 8.5 and 23 °C (a), 30 °C (b), and 37 °C (c) for 24 and 48 h in the presence of DTT.M, molecular mass markers; lane 1, purified Var-GyrA after anion IEX; lane 2, cleavage with 5 mM DTT for 24 h; lane 3, cleavage with 10 mM DTT for 24 h; lane 4, cleavage with 25 mM DTT for 24 h; lane 5, cleavage with 50 mM DTT for 24 h; lane 6, cleavage with 5 mM DTT for 48 h; lane 7, cleavage with 10 mM DTT for 48 h; lane 8, cleavage with 25 mM DTT for 48 h; lane 9, cleavage with 50 mM DTT for 48 h. 15%-SDS-PAGE. Arrows indicate the Var-GyrAfusion protein (A) and residual protein CBD-GyrA (B).Table S2. Material balance of the haemadin purification. Table S3. Material balance of the variegin purification. Figure S5. Dose–response curve of inhibition of amidolytic thrombin activity by hirudin-1 (a), haemadin (b), anophelin (c), and variegin (d) without pre-incubation (slow-binding inhibition) and after 30 min incubation (tight-binding inhibition) with thrombin. All assays were performed with 500 pM thrombin, 100 µM S-2238, and different concentrations of the inhibitor. * indicates *p* ≤ 0.05 for IC50 values relative to the 30 min incubation values according to a Kruskal–Wallis test. Table S4. Effect on APTT in tests with mouse plasma. Table S5. Effect on PT in tests with mouse plasma. Table S6. Effect on TT in tests with mouse plasma. Table S7. Effect on APTT in tests with human plasma. Table S8. Effect on PT in tests with human plasma. Table S9. Effect on TT in tests with human plasma.
