# Supplementary material for: Screening of the Promising Direct Thrombin Inhibitors from Haematophagous Organisms. Part I: Recombinant Analogues and Their Antithrombotic Activity In Vitro
Source: Biomedicines. 2021 Dec 22;10(1):11. doi: 10.3390/biomedicines10010011 (PMC8772750; doi:10.3390/biomedicines10010011)
Supplement: Supplementary file 1 [file biomedicines-10-00011-s001.zip › biomedicines-1488266-supplementary.pdf]

Supplementary Material

Screening of the promising direct thrombin inhibitors from haematophagous organisms. Part I. Antithrombotic activity in vitro.

Maria A. Kostromina[1*], Elena A. Tukhovskaya[2*], Elvira R. Shaykhutdinova[2], Gulsara A. Slashcheva[2], Alina M. Ismailova[2], Victor A. Palikov[2], Yuliya A. Palikova[2], Igor A. Dyachenko[2], Irina N. Kravchenko[2], Elena S. Sadovnikova[2], Nadezhda I. Novikova[2], Natalia A. Perepechenova[3], Evgeniy A. Zayats[1], Yuliya A.Abramchik[1], Dmitry D. Lukoshin[1], Andrey N. Mamaev[4], Elena V. Grigorieva[4], Andrey P. Momot[4], Arkady N. Murashev[2], Roman S. Esipov[1]

[1] Shemyakin-Ovchinnikov Institute of Bioorganic Chemistry, Russian Academy of Sciences, 16/10 Miklukho-Maklaya St., 117997 Moscow, Russia

[2] Biologycal Testing Laboratory, Affiliated Branch of M. M. Shemyakin and Yu. A. Ovchinnikov Institute of Bioorganic Chemistry, Russian Academy of Sciences, Pushchino, Russia

[3] Laboratory of toxicology in vitro, Affiliated Branch of M. M. Shemyakin and Yu. A. Ovchinnikov Institute of Bioorganic Chemistry, Russian Academy of Sciences, Pushchino, Russia

[4] Altai Branch of the National Research Center for Hematology, 656045, Barnaul, Russian Federation

Table S1. Oligonucleotide primers used to construct synthetic haemadin and variegin genes.

Primer	5'–3' sequence	Restriction Enzymes
**Haemadin**		
**Hae-Prim**	GGTGGTTGCTCTTCCAACATTCGCTTCGG	LguI
**Hae1**	GGTGGTTGCTCTTCCAACATTCGCTTCGGCATGGGCAAAGTGCCG	LguI
**Hae2**	TGCCCGGATGGCGAAGTGGGCTATACCTGCGATTGCGGCG	
**Hae3**	AAAAAATTTGCCTGTATGGCCAGTCTTGCAACGATGGCCAGTGCTCT	
**Hae4**	GGCGATCCGAAACCGTCTTCTGAATTCGAAGAGTTCGAGATTGATGAA	
**Hae5**	GGAGAAA**TGA**TTAGGATCCACCACC	
**Hae6**	GGTGGTGGATCCTAATCATTTCTCCTCTTCATCAATCTCGAACTCTTCGAAT	BamHI
**Hae7**	TCAGAAGACGGTTTCGGATCGCCAGAGCACTGGCCATCGTTGCA	
**Hae8**	AGACTGGCCATACAGGCAAATTTTTTCGCCGCAATCGCAGGTATAGC	
**Hae9**	CCACTTCGCCATCCGGGCACGGCACTTTGCCCATGCCGAA	
**Hae10**	GCGAATGTTGGAAGAGCAACCACC	
**Hae-Revs**	GGTGGTGGATCCTAATCATTTCTCCTCTTCATCA	BamHI
**Variegin**		
**Var-Prim**	GGTGGTCAT**ATG**AGCGACCAGGGCGAC	NdeI
**Var1**	GGTGGTCAT**ATG**AGCGACCAGGGCGACGTGGCGGAACCGAAAATGCATA	NdeI
**Var2**	AAACCGCGCCGCCGTTTGATTTTGAAGCGATTCCGGAAGAATATCTGGACG	
**Var3**	ACGAAAGCTGCGGAAGAGCAACCACC	
**Var4**	GGTGGTTGCTCTTCCGCAGCTTTCGTCGTCCAGATATTCTTCCGGAATCGCTTCA	LguI
**Var5**	AAATCAAACGGCGGCGCGGTTTTATGCATTTTCGGTTCCGCCACGTC	
**Var1**	GCCCTGGTCGCTCATATGACCACC	
**Var-Revs**	GTGGTTGCTCTTCCGCAGCTTTCGTC	LguI

LguI and BamHI recognition sites are underlined in the primers for construction of haemadin gene. NdeI and BamHI recognition sites are underlined in the primers for construction of variegin gene. The start and stop codons are boldfaced.

Haemadin IRFGMGKVPCPDGEVGYTCDCGEKICLYGQSCNDGQCSGDPKPSSEFEEFEIDEEEK

Variegin SDQGDVAEPKMHKTAPPFDFEAIPEEYLDDES

(**a**)

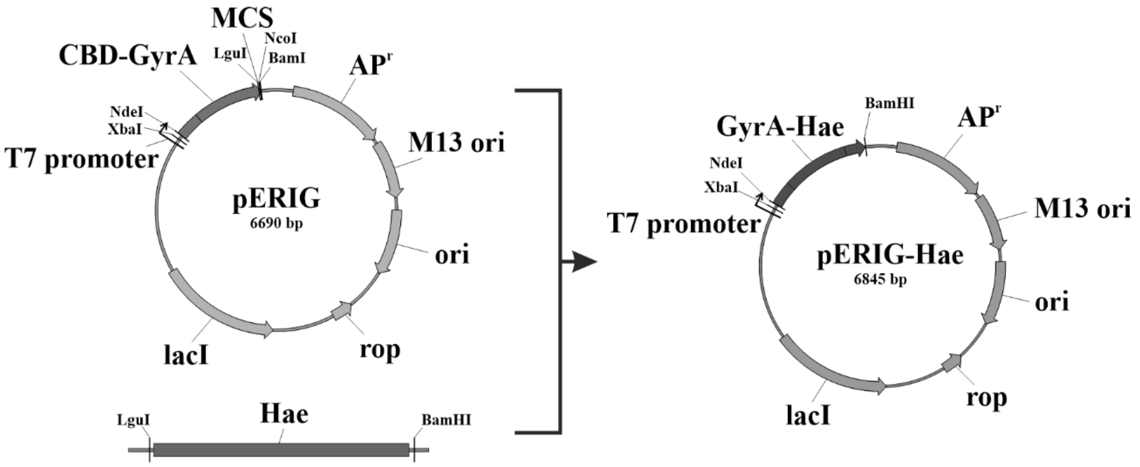

(**b**)

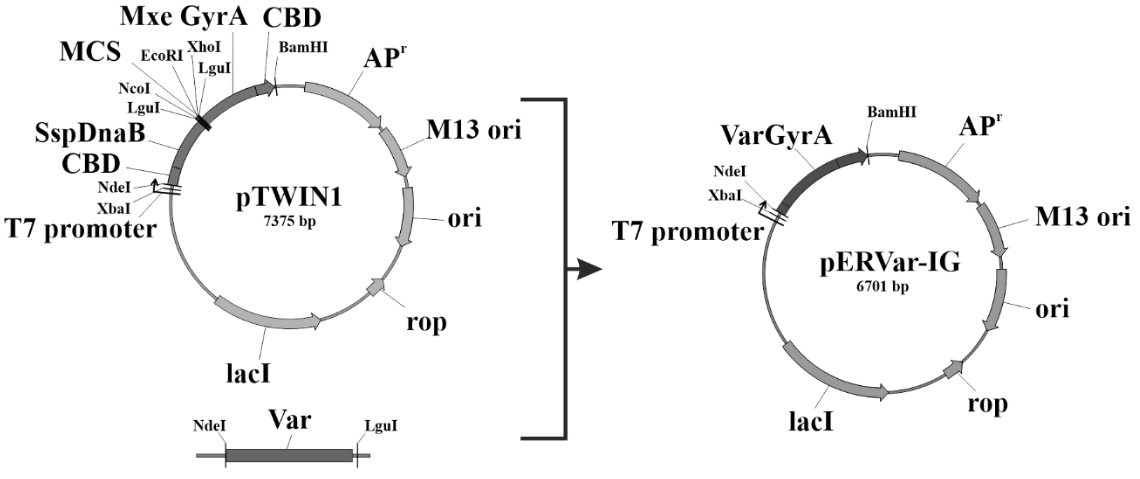

(**c**)

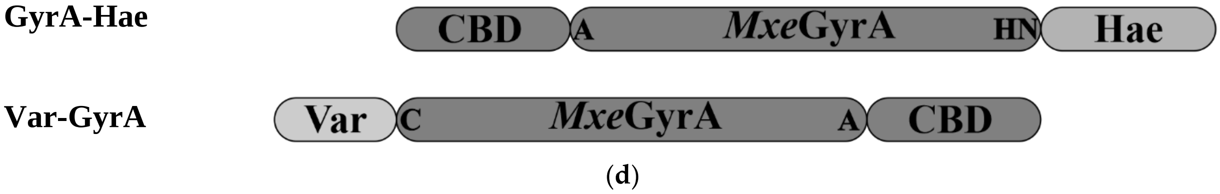

(**d**)

Figure S1. (**a**) Amino acid sequences of haemadin and variegin. (**b**) Construction scheme of expression vector pERIG-Hae. (**c**) Construction scheme of expression vector pERVar-IG. (**d**) GyrA-Hae and Var-GyrA fusion proteins. CBD, chitin-binding domain; *Mxe*GyrA, mini-intein from *Mycobacterium xenopi*; A, alanine; H, histidine; N, asparagine; C, cysteine.

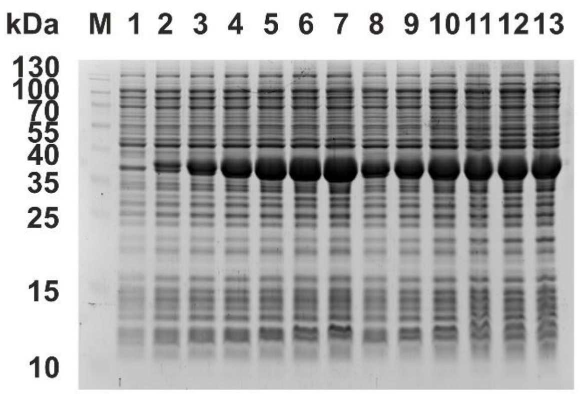

(**a**)

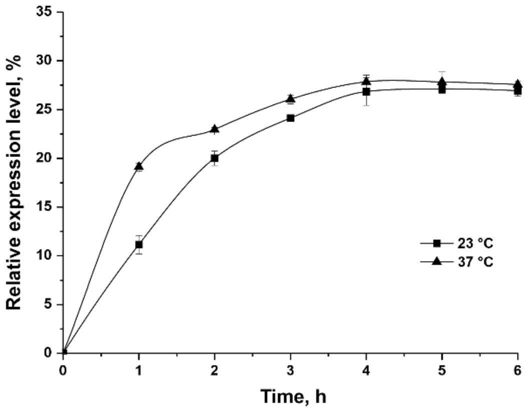

(**b**)

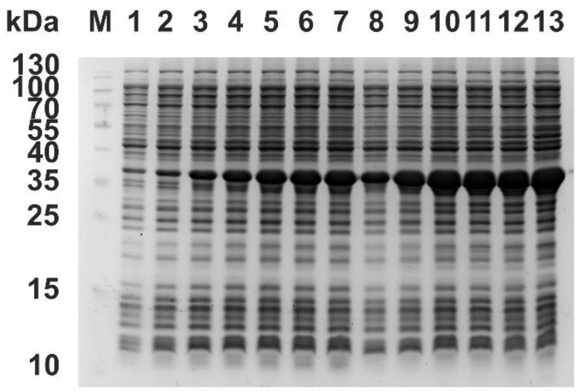

(**c**)

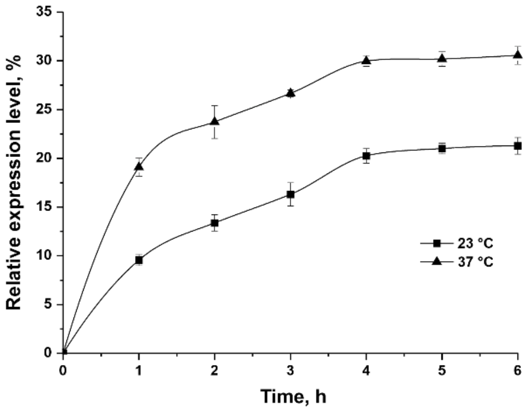

(**d**)

Figure S2. Optimization of cultivation conditions of the producer strain *E. coli* BL21(DE3)/pERIG-Hae (**a, b**) and *E. coli* BL21(DE3)/pERVar-IG (**c, d**). (**a, c**) The SDS-PAGE analysis of fusion protein GyrA-Hae (**a**)and Var-GyrA (**c**) accumulation during the producer strains cultivation. M, molecular mass markers; Lane 1, uninduced crude cell extract; lanes 2–7, crude cell extracts after induction for 1 – 6 h at 23 °C; lanes 8–13, crude cell extracts after induction for 1 – 6 h at 37 °C. 15 %-SDS-PAGE **b, d.** Dynamics of fusion protein GyrA-Hae (**b**) and Var-GyrA (**d**) accumulation during the producer strains cultivation. * $p \leq 0.05$ relative to 37 C °C according to a Mann–Whitney U test.

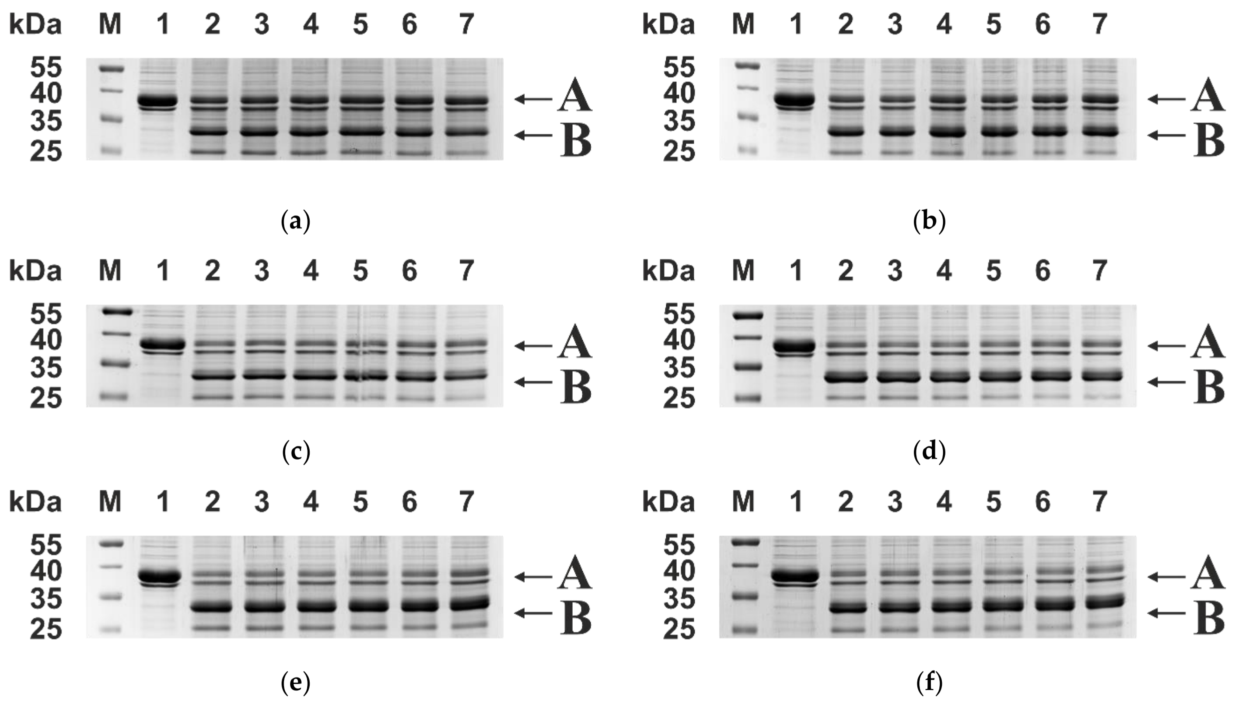

Figure S3. Optimization of GyrA-Hae fusion protein cleavage. **(a, c, e)** cleavage after 24-h incubation in the range pH 6.0 to 7.0 at 23 °C (**a**), 30 °C (**b**) and 37 °C (**e**). **(b, d, f)** cleavage after 48-h incubation in the range pH 6.0 to 7.0 at 23 °C (**b**), 30 °C (**d**) and 37 °C (**f**). M, molecular mass markers; lane 1, purified GyrA-Hae after anion IEX; lane 2, cleavage at pH 6.0; lane 3, cleavage at pH 6.2; lane 4, cleavage at pH 6.4; lane 5, cleavage at pH 6.6; lane 6, cleavage at pH 6.8; lane 7, cleavage at pH 7.0. 15 %-SDS-PAGE. Arrows indicate GyrA-Hae fusion protein (A) and residual protein CBD-GyrA (B).

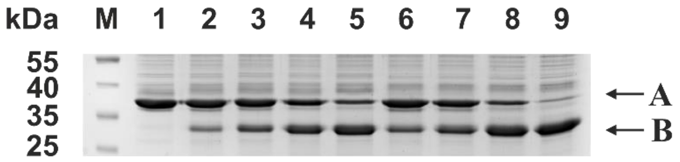

(**a**)

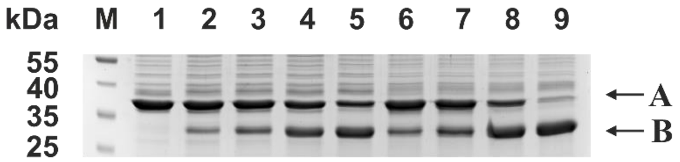

(**b**)

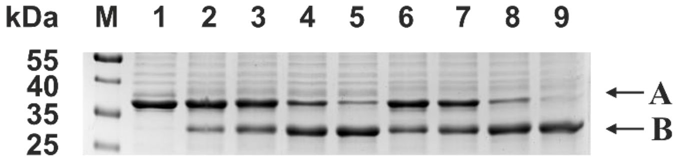

(**c**)

Figure S4. Optimization of Var-GyrA fusion protein cleavage at pH 8.5 and 23 °C (**a**), 30 °C (**b**) and 37 °C (**c**) for 24 and 48 h in the presence of DTT. M, molecular mass markers; lane 1, purified Var-GyrA after anion IEX; lane 2, cleavage with 5 mM DTT for 24 h; lane 3, cleavage with 10 mM DTT for 24 h; lane 4, cleavage with 25 mM DTT for 24 h; lane 5, cleavage with 50 mM DTT for 24 h; lane 6, cleavage with 5 mM DTT for 48 h; lane 7, cleavage with 10 mM DTT for 48 h; lane 8, cleavage with 25 mM DTT for 48 h; lane 9, cleavage with 50 mM DTT for 48 h. 15 %-SDS-PAGE. Arrows indicate Var-GyrA fusion protein (A) and residual protein CBD-GyrA (B).

Table S2. Material balance of the haemadin purification

Step of purification	Total protein	GyrA-Hae fusion protein		Haemadin		Step yields	Relative product yield
	mg [2]	mg [3]	% [4]	mg [5]	% [6]	% [7]	% [8]
**Cell supernatant [1]**	9487	2637	27.80	ND	ND	100.00	100.00
**Anion IEX on Q Sepharose XL**	4313	2519	58.40	ND	ND	95.51	95.51
**Fusion protein cleavage**	4313	565	13.10	537	12.45	86.90	74.09
**Anion IEX on Q Sepharose HP**	583	–	–	521	89.30	97.02	71.88
**RP-HPLC**	515	–	–	508	98.70	97.50	70.08
**Size-exclusion chromatography**	507	–	–	502	98.92	98.82	69.26
**Lyophilization**	503	–	–	497	98.90	99.00	68.57

[1] From 100 g of of cell biomass.
[2] Protein concentration determined by Lowry assay using BSA as a standard protein.
[3] The GyrA-Hae fusion protein amount was calculated as the GyrA-Hae fusion protein content multiplied by the amount of total protein.
[4] The GyrA-Hae fusion protein content was measured using densitometric analysis of proteins on SDS-PAGE.
[5] The haemadin amount was calculated using the calibration curve plotted based on RP-HPLC analysis of haemadin standard solution.
[6] The haemadin purity was calculated as the haemadin amount divided by the amount of total protein (defined as 100%).
[7] Step yields was calculated as the amount of target protein at that step (fusion protein at the steps 1 – 3 and haemadin at the steps 4 – 7) divided by the amount of target in the previous step (defined as 100%).
[8] Relative product yield was calculated by multiplying the yields of each previous process steps
ND – not determined

Table S3. Material balance of the variegin purification

Step of purification	Total protein	Var-GyrA fusion protein		Variegin		Step yields	Relative product yield
	mg [2]	mg [3]	% [4]	mg [5]	% [6]	% [7]	% [8]
**Cell supernatant** [1]	9007	2954	32.80	ND	ND	100.00	100.00
**Anion IEX on Q Sepharose XL**	4779	2982	62.40	ND	ND	100.94	100.94
**Fusion protein cleavage**	4779	292	6.10	239	4.94	93.90	91.07
**Anion IEX on Q Sepharose HP**	292	–	–	224	76.70	93.72	85.36
**RP-HPLC**	209	–	–	207	99.38	92.41	78.88
**Size-exclusion chromatography**	196	–	–	194	99.31	93.72	73.92
**Lyophilization**	191	–	–	189	99.35	97.42	72.02

[1] From 100 g of of cell biomass.
[2] Protein concentration determined by Lowry assay using BSA as a standard protein.
[3] The Var-GyrA fusion protein amount was calculated as the Var-GyrA fusion protein content multiplied by the amount of total protein.
[4] The Var-GyrA fusion protein content was measured using densitometric analysis of proteins on SDS-PAGE.
[5] The variegin amount was calculated using the calibration curve plotted based on RP-HPLC analysis of variegin standard solution.
[6] The variegin purity was calculated as the variegin amount divided by the amount of total protein (defined as 100%).
[7] Step yields was calculated as the amount of target protein at that step (fusion protein at the steps 1 – 3 and variegin at the steps 4 – 7) divided by the amount of target in the previous step (defined as 100%).
[8] Relative product yield was calculated by multiplying the yields of each previous process steps
ND – not determined

Inhibition of the amidolytic thrombin activity

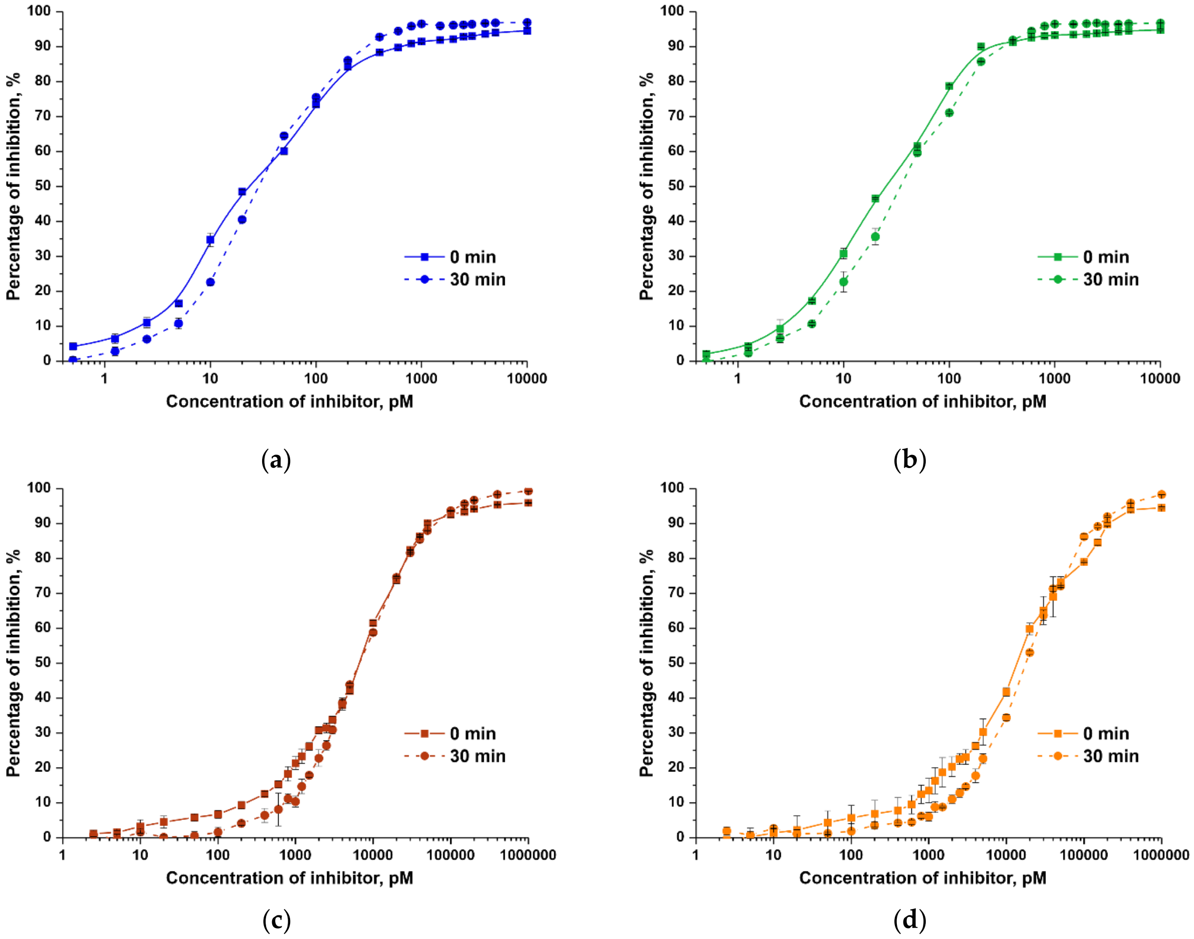

Figure S5. Dose-response curve of inhibition of amidolytic thrombin activity by hirudin-1 (**a**), haemadin (**b**), anopheline (**c**) and variegin (**d**) without preincubation (slow-binding inhibition) and after 30 min incubation (tight-binding inhibition) with thrombin. All assays were performed with 500 pM thrombin, 100 µM S-2238 and different concentration of inhibitor.

* p≤0.05 for IC50 relative to 30 min incubation values according to Kruskal Wallis test

Effect on clotting times in test with mouse plasma

Table S4. Effect on APTT in test with mouse plasma

Dose	Hirudin-1	Haemadin	Anophelin	Variegin	PBS
0.1μg/mL	25.6 ± 3.3	26.4 ± 2.2	25.6 ± 1.4	28.6 ± 5.2	23.8 ± 1.5
1 μg/mL	40.8 ± 7.1	45.1 ± 7.3	27.6 ± 3.0	42.2 ± 10.5	
10 μg/mL	99.8 ± 11.4 **##	119.9 ± 15.6**##	40.6 ± 3.8 *#	142.2 ± 12.1***	
100 μg/mL	Above 180	Above 180	112.1 ± 24.7 ***	Above 180	

n = 6 for each group
* p≤0.05, ** p≤0.01, *** p≤0.001 relative to the Saline group (taken as the zero point for each group) according to the Kruskal Wallis test
# p≤0.05, ## p≤0.01 relative to the Dose 0.1 μg/mL group according to the Kruskal Wallis test

Table S5. Effect on PTT in test with mouse plasma

Dose	Hirudin-1	Haemadin	Anophelin	Variegin	PBS
0.1μg/mL	10.4 ± 0.3	10.0 ± 1.0	9.6 ± 0.3	11.4 ± 0.6	11.2 ± 0.6
1 μg/mL	11.6 ± 0.9	10.5 ± 0.5	9.8 ± 1.1	13.2 ± 0.8	
10 μg/mL	63.2 ± 5.3 ##	Above 180	11.4 ± 0.9#	44.9 ± 8.3 *##	
100 μg/mL	Above 180	Above 180	Above 180	Above 180	

n = 6 for each group
* p≤0.05 relative to the Saline group (taken as the zero point for each group) according to the Kruskal Wallis test;
# p≤0.05,## p≤0.01 relative to the Dose 0.1μg/mL group according to the Kruskal Wallis test

Table S6. Effect on TT in test with mouse plasma

Dose	Hirudin-1	Haemadin	Anophelin	Variegin	PBS
0.1μg/mL	22.4 ± 1.8	11.1 ± 0.4**	23.1 ± 2.5**	33.4 ± 4.7	21.8 ± 2
1 μg/mL	Above 180	Above 180	86.3 ± 6.9*	109.3 ± 6.1***	
10 μg/mL	Above 180	Above 180	Above 180	Above 180	
100 μg/mL	Above 180	Above 180	Above 180	Above 180	

n = 6 for each group
* p≤0.05,** p≤0.01, *** p≤0.001 relative to the Saline group (taken as the zero point for each group) according to the Kruskal Wallis test;

Effect on clotting times in test with human plasma

Table S7. Effect on APTT in test with human plasma

Dose	Hirudin-1	Haemadin	Anophelin	Variegin	PBS
0.01 μg/mL	44.5 ± 0.6	62.1±1.3	41.5±0.5	52.6±0.8$^{@@}$	38.4±0.2
0.1 μg/mL	57.2 ± 2.0 *	56.2 ± 1.2$^{@@}$	41.1 ± 0.5$^{@}$	55.8 ± 0.8	
1 μg/mL	96.1 ± 2.3 **#	90.4 ± 1.8**	85.9 ± 0.7**	86.2 ± 1.4**	
10 μg/mL	Above 180	173.2 ± 5.0**	85.9 ± 0.7**	167.4 ± 2.5**	

n = 6 for each group
* p≤0.05,** p≤0.01 relative to the Saline group (taken as the zero point for each group) according to the Kruskal Wallis test;
# p≤0.05 relative to the Dose 0.01μg/mL group according to the Kruskal Wallis test;
@ p≤0.05,@@ p≤0.01 relative to the Dose 10 μg/mL group according to the Kruskal Wallis test

Table S8. Effect on PTT in test with human plasma

Dose	Hirudin-1	Haemadin	Anophelin	Variegin	PBS
0.01 μg/mL	13.3 ± 0.1$^{@@}$	13.8 ± 0.1	13.6 ± 0.2	14.1 ± 0.2	15.7 ± 0.4
0.1 μg/mL	14.2 ± 0.2	13.6 ± 0.2$^{@}$	13.2 ± 0.2$^{@}$	14.0 ± 0.2$^{@}$	
1 μg/mL	16.0 ± 0.2*	15.1 ± 0.2*	18.6 ± 0.2	17.1 ± 0.4*	
10 μg/mL	44.2 ± 1.2**	28.0 ± 0.3**	18.6 ± 0.2**	46.1 ± 1.5**	

n = 6 for each group
* p≤0.05,** p≤0.01 relative to the Saline group (taken as the zero point for each group) according to the Kruskal Wallis test;
@ p≤0.05,@@ p≤0.01 relative to the Dose 10 μg/mL group according to the Kruskal Wallis test

Table S9. Effect on TT in test with human plasma

Dose	Hirudin-1	Haemadin	Anophelin	Variegin	PBS
0.01 μg/mL	27.1 ± 1.0*	23.3 ± 0.6*	20.7 ± 0.3*	20.2 ± 0.4	26.4 ± 0.2
0.1 μg/mL	21.6 ± 0.5	23.5 ± 0.1*	20.4 ± 0.2	23.1 ± 0.9*	
1 μg/mL	Above 180	Above 180	Above 180	102.5 ± 2.1***	
10 μg/mL	Above 180	Above 180	Above 180	Above 180	

n = 6 for each group
* p≤0.05,*** p≤0.001relative to the Saline group (taken as the zero point for each group) according to the Kruskal Wallis test
